# Use of H1N1 strain A/PR/8/34 influenza to build a mouse model of viral respiratory sepsis

**DOI:** 10.1186/s42826-025-00248-4

**Published:** 2025-06-04

**Authors:** Yaqing Jiao, Yuee Cai, Yilin Zhang, Ka-Tim Choy, Ka-Man Cheng, John M. Nicholls, Pui-Kin Lam, Hui-Ling Yen, Timothy H. Rainer

**Affiliations:** 1 Department of Emergency Medicine, Li Ka Shing Faculty of Medicine, School of Clinical Medicine, The University of Hong Kong, Hong Kong Special Administrative Region, China; 2 School of Public Health, Li Ka Shing Faculty of Medicine, The University of Hong Kong, Hong Kong Special Administrative Region, China; 3 Department of Pathology, Li Ka Shing Faculty of Medicine, The University of Hong Kong, Hong Kong, China

**Keywords:** Influenza virus, Mouse model, Organ dysfunction, Viral respiratory sepsis

## Abstract

**Background:**

Community-acquired respiratory infections are a prevalent cause of sepsis. Current animal models simulate peritoneal rather than respiratory sepsis. This study sought to appraise an influenza model for its ability to develop sepsis.

**Methods:**

Twenty-four six-week-old male BALB/c mice were intranasally inoculated with H1N1 strain A/PR/8/34 virus at 3.7 × 10^− 1^, 3.7 × 10^0^, 3.7 × 10^1^, 3.7 × 10^2^, 3.7 × 10^3^, 3.7 × 10^4^ median tissue culture infectious dose (TCID50) to acquire different levels of clinical severity. Murine Sepsis Score (MSS) was recorded daily over 14 days. Platelets, serum bilirubin and creatinine levels were measured to reflect coagulopathy, liver and renal dysfunction. These three parameters are from the Sequential Organ Failure Assessment (SOFA) score which is routinely used for monitoring human sepsis. The primary outcome is organ dysfunction.

**Results:**

Out of 24 infected mice, seven (29%) did not survive beyond 9 days. MSS predicted mortality with an AUC of 0.989 (95%CI: 0.978-1.000; *P* < 0.001). Liver and renal dysfunction were detected in one non-survived and six survived mice. Histological examination revealed inflammation in lung and liver but not kidney tissues.

**Conclusions:**

This study demonstrates the potential of influenza to cause organ dysfunction, providing a basis for building a murine model specific for viral respiratory sepsis, and more closely simulating human viral sepsis.

**Supplementary Information:**

The online version contains supplementary material available at 10.1186/s42826-025-00248-4.

## Background

Sepsis is a life-threatening organ dysfunction caused by a dysregulated host response to infection [1]. Sepsis affects 49 million people annually worldwide, with 25% of hospital cases resulting in death [2, 3]. Community-acquired respiratory infection is one prevalent cause of sepsis in emergency departments [4]. Viral aetiology accounts for 30–40% of sepsis but remains understudied [5–7]. Influenza affects a billion people, causing 290,000 to 650,000 respiratory deaths yearly [8]. Most respiratory virus fatalities end in sepsis [9].

Current experimental models simulate peritoneal sepsis, including injection of lipopolysaccharide (LPS), injection of live pathogens, and impairment of barrier tissue integrity [10, 11]. There is no good murine model for viral respiratory sepsis, which may have contributed to translational failures of applying preclinical findings to human trials.

Murine models infected with viruses are widely studied but rarely assessed from the perspective of sepsis [12, 13]. Severe, lethal respiratory virus models may develop sepsis before death. This study assessed sepsis in a pneumonia model using the mouse-adapted, influenza virus A/PR/8/34 (H1N1). We aimed to build a model that is specific for viral respiratory sepsis.

## Methods

Six-week-old male BALB/c mice (20–25 g) were intranasally inoculated with H1N1 strain A/PR/8/34 virus at doses of 3.7 × 10^− 1^, 3.7 × 10^0^, 3.7 × 10^1^, 3.7 × 10^2^, 3.7 × 10^3^ and 3.7 × 10^4^ median tissue culture infectious dose (TCID50) (*n* = 4 per dose) to acquire infections with different levels of clinical severity. The workflow is shown in Supplementary file 1. The sample size aligns with the protocol used to determine the median lethal dose (MLD50) of this virus and was repurposed for this study. Sham mice received PBS (*n* = 3). Mice were monitored daily for survival, weight, glucose, and clinical conditions via Murine Sepsis Score (MSS) [14, 15]. MSS evaluates severity based on appearance, level of consciousness, activity, response to stimulus, eye opening, respiratory rate and respiratory quality (Supplementary file 2). Mice were euthanised when they met our institutional humane endpoints (HEP) for animal euthanasia or at the end of the experiment (14 days). HEP were > 20% weight loss, or MSS > 10. Blood samples were collected immediately prior to euthanasia, allowing correlation of biochemical markers with MSS peaks, weight loss, and survival trends. Platelets, bilirubin and creatinine were quantified to reflect coagulopathy, liver and renal dysfunction, aligning with the Sequential Organ Failure Assessment (SOFA) score which is routinely used for monitoring human sepsis (Supplementary file 3) [1, 16]. Whole blood platelet count was measured using a Sysmex XN-1000 V haematology analyser, and serum bilirubin and creatinine using a Sysmex BX-3010 chemistry analyser (Sysmex, Norderstedt, Germany). Lung, liver, kidney, spleen, brain and heart tissues were examined by histology as reported previously [17].

All analyses and figures were computed with GraphPad Prism (v.10 GraphPad Software, USA). Statistical significance was determined by Mann–Whitney U test, t test, and Kruskal–Wallis test. *P* < 0.05 was considered as statistically significant. Data were presented as median with 95% confidence interval (CI) and mean with standard deviation (SD). The correlation analysis was conducted by Spearman’s rank test. We report findings according to the ARRIVE (Animal Research: Reporting of In Vivo Experiments) guidelines 2.0 [18].

## Results

### MSS reflects physical conditions and predicts death after H1N1 infection

#### Survival rate

**Fig. 1:**
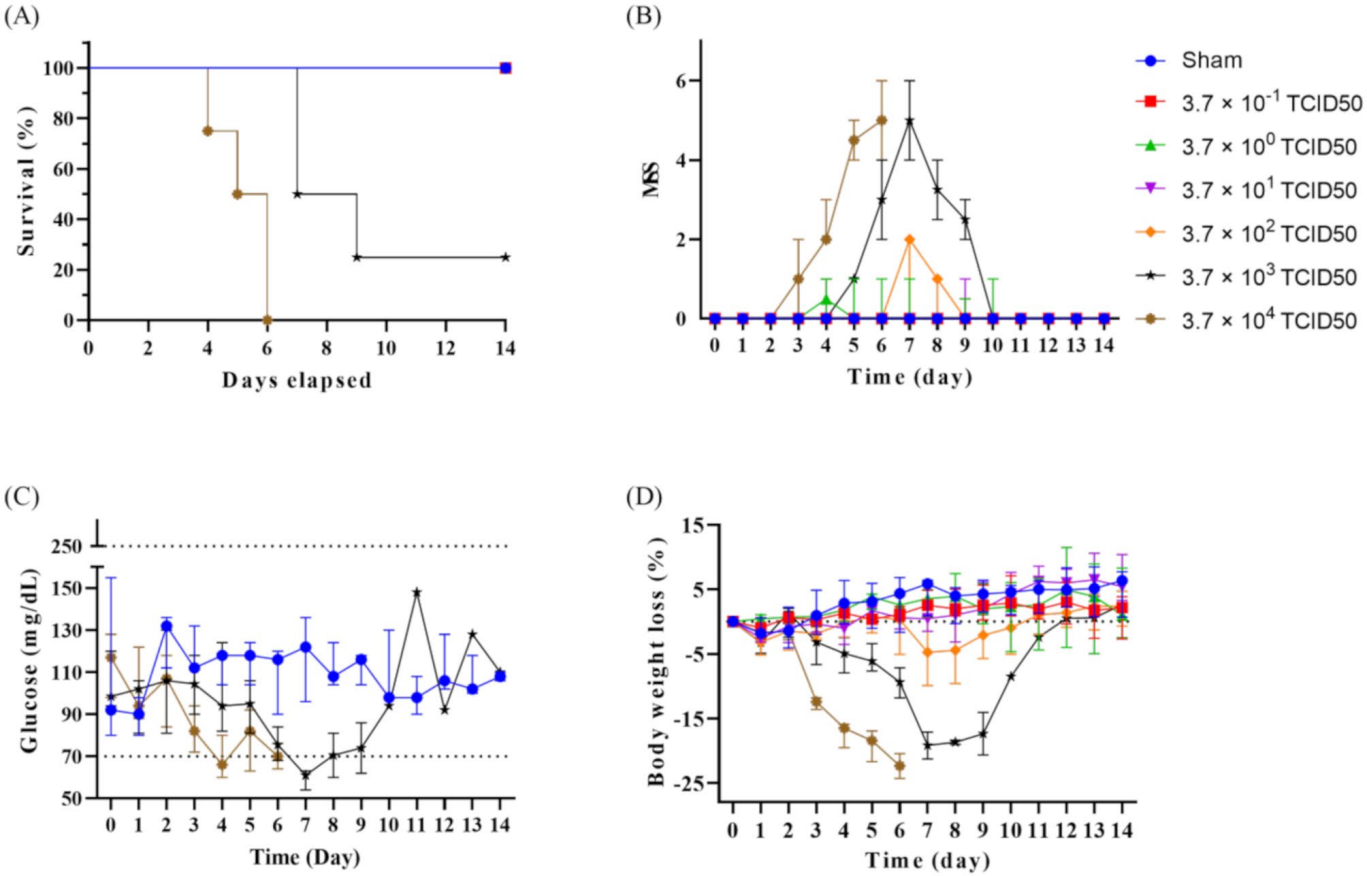
Physical conditions of 6-week-old male BALB/c mice after intranasally inoculated with influenza virus H1N1 strain A/PR/8/34. Doses of at 3.7 × 10^− 1^, 3.7 × 10^0^, 3.7 × 10^1^, 3.7 × 10^2^, 3.7 × 10^3^ and 3.7 × 10^4^ median tissue culture infectious dose (TCID50) were evaluated over 14 days. Survival curve (**A**), Murine Sepsis Score (MSS) (**B**), blood glucose level (**C**), and body weight loss (**D**). Sham (PBS control): *n* = 3; H1N1 per dose group: *n* = 4. Data are presented as median with 95% confidence interval (CI)

Of 24 infected mice, seven (29%) did not survive beyond nine days. After being inoculated with 3.7 × 10^4^ TCID50, one mouse reached euthanasia criteria on day 4, and survival rate by day 6 was 0%. After being inoculated with 3.7 × 10^3^ TCID50, only one mouse survived beyond 14 days with a survival rate of 25% at day 9 (Fig. 1A). No deaths occurred at lower doses.

#### MSS

**Fig. 2:**
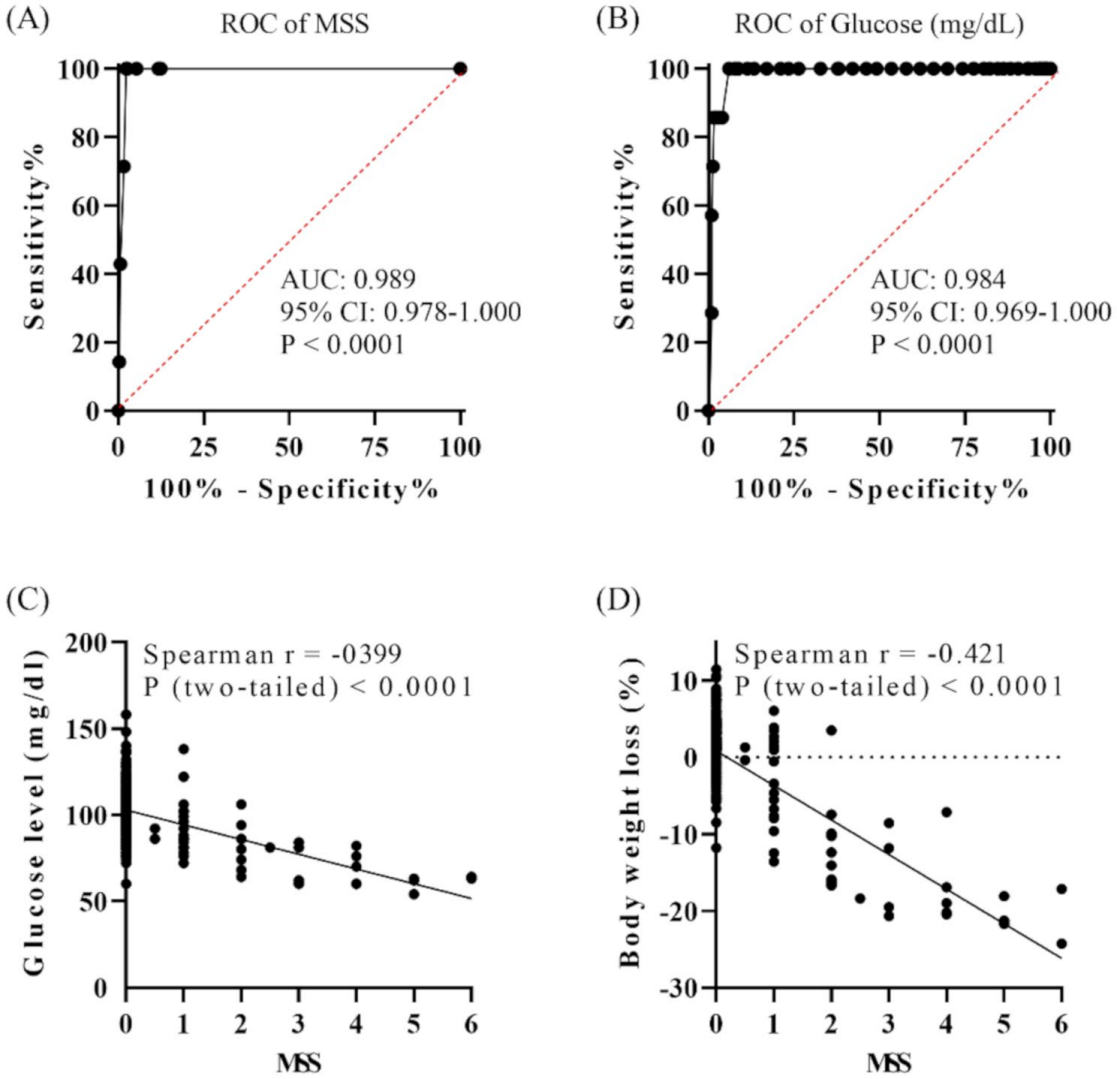
Performance of the Murine Sepsis Score (MSS) and blood glucose level in differentiating influenza virus H1N1 strain A/PR/8/34 infected mice that survived and non-survived over 14 days. Receiver Operating Characteristic (ROC) curves of MSS (**A**) and blood glucose level (**B**). Correlation between MSS and blood glucose level (**C**), MSS and body weight loss (**D**). H1N1 infected that survived: *n* = 17 and non-survived: *n* = 7. AUC: The area under the ROC curve. 95% CI: 95% confidence interval

Mice monitored by MSS exhibited abnormal symptoms starting day 3, peaking at day 7 (maximum MSS of 6), and recovering by day 10 (Fig. 1B). Higher doses were associated with higher MSS, matching mortality rate. MSS effectively predicted death in H1N1-infected mice, with an AUC of 0.989 (95% CI: 0.978–1.000) (Fig. 2A). At a cut-off score of > 2.75, sensitivity was 100% (95% CI: 59.04–100%), and specificity was 97.65% (95% CI: 95.22–99.05%).

#### Glycaemia

As blood glucose levels fluctuate in patients with sepsis, we assessed blood glucose variation in the infected mice [19, 20]. Infected mice exhibited hypoglycaemia before death, glucose dropping below 70 mg/dl (Fig. 1C). The AUC for glucose predicting mortality was 0.984 (95% CI: 0.969–1.000) (Fig. 2B). At a cut-off of < 77 mg/dl, sensitivity was 100% (95% CI: 59.04–100%), and specificity was 93.98% (95% CI: 90.65–96.30%).

#### Body weight loss

Surviving mice experienced maximum weight loss on day 7 before recovery (Fig. 1D). A 20% body weight loss met HEP criteria for euthanasia. All non-survived mice reached this criterion before euthanasia instead of natural death.

Correlations between MSS and glucose levels, and MSS and weight loss were 0.399 and 0.421, respectively (Spearman rank *P* < 0.0001) (Fig. 2C and D). MSS reflects physical conditions and predicts death after H1N1 infection.

### Organ dysfunction does not necessarily lead to death, and vice versa

#### Bilirubin

**Fig. 3:**
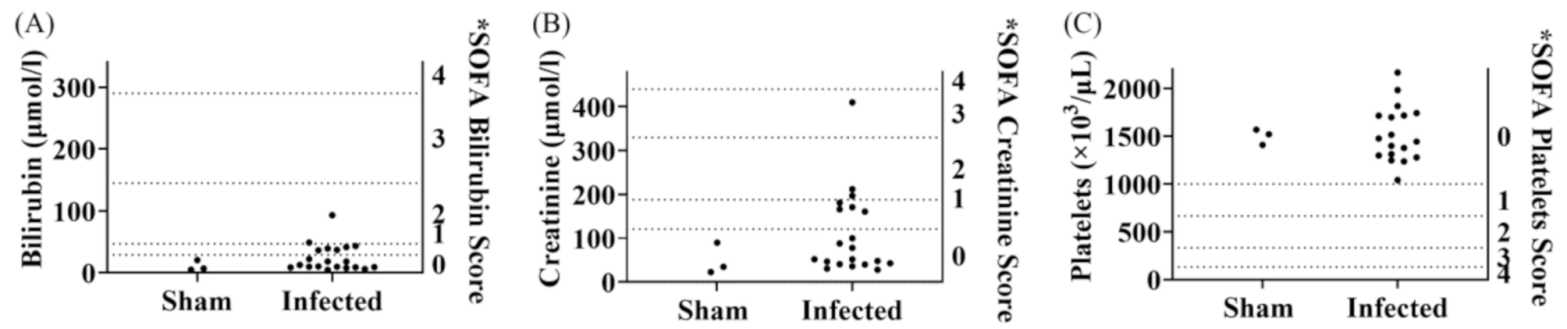
Application of SOFA score on sham (PBS control) and influenza virus H1N1 strain A/PR/8/34 infected mice. *SOFA score of mouse serum bilirubin (**A**), creatinine (**B**), and platelets (**C**). *: Each criterion in the SOFA score was proportionally assigned to score the respective mouse parameters. Sham (PBS control): *n* = 3; H1N1 infected with available biochemistry data: *n* = 20

SOFA bilirubin reflects liver function. Normal serum bilirubin in mice was reported as 1.71 to 18.81 µmol/L [21]. This range can vary depending on factors such as strain, age, gender and specific study conditions. In this study, sham mice had a mean of 10.82 µmol/L (SD 8.59). Thus, the normal range (Mean ± 2 SD) was calculated as 0–28.00 µmol/L. Seven infected mice had bilirubin levels of 36.49 to 93.10 µmol/L, outside normal limits. Human SOFA normal range for this variable is 0–20 µmol/L, scored as 0 [16]. By proportion, sham mice and thirteen infected mice scored 0, five scored 1, and two scored 2 (Fig. 3A; Supplementary file 4 and 5). Of seven mice with scores ≥ 1, only one died by day 7, and other six survived beyond 14 days. There was no dose-response relationship between serum bilirubin levels and virus doses.

#### Creatinine

SOFA creatinine reflects renal function. Normal mouse creatinine was reported as 17.68 to 159.12 µmol/L [21]. In this study, sham mice had a mean of 49.33 µmol/L (SD 35.73), giving a calculated normal range of 0–120.79 µmol/L, matching human SOFA score 0 (0–110 µmol/L) for this variable [16]. By proportion, four infected mice scored 1, two scored 2, and one scored 3 (Fig. 3B; Supplementary file 4 and 5). Seven mice scoring creatinine ≥ 1 also scored bilirubin ≥ 1, indicating liver and renal dysfunction occurred simultaneously.

#### Platelets

Sham mice had a mean platelet count of 1,499 × 10^3^/µL (SD 82.24), with a calculated normal range of 1,335–1,663 × 10^3^/µL. Previous studies reported normal mouse platelet counts as 1,000–1,500 × 10^3^/µL, and in human as 150–400 × 10^3^/µL [22]. Human SOFA platelet score of 0 corresponds to platelets ≥ 150 × 10^3^/µL [16]. All the infected mice scored 0, with a mean platelet count of 1,526 × 10^3^/µL (SD 292.1), showing no significant differences from sham controls (*P* = 0.8779; t test) (Fig. 3C; Supplementary file 4 and 5). There was no coagulopathy in the infected mice. MSS does not predict organ dysfunction (AUC *P* = 0.4556).

### Tissue damage in lung and liver were identified in the H1N1 model

**Fig. 4:**
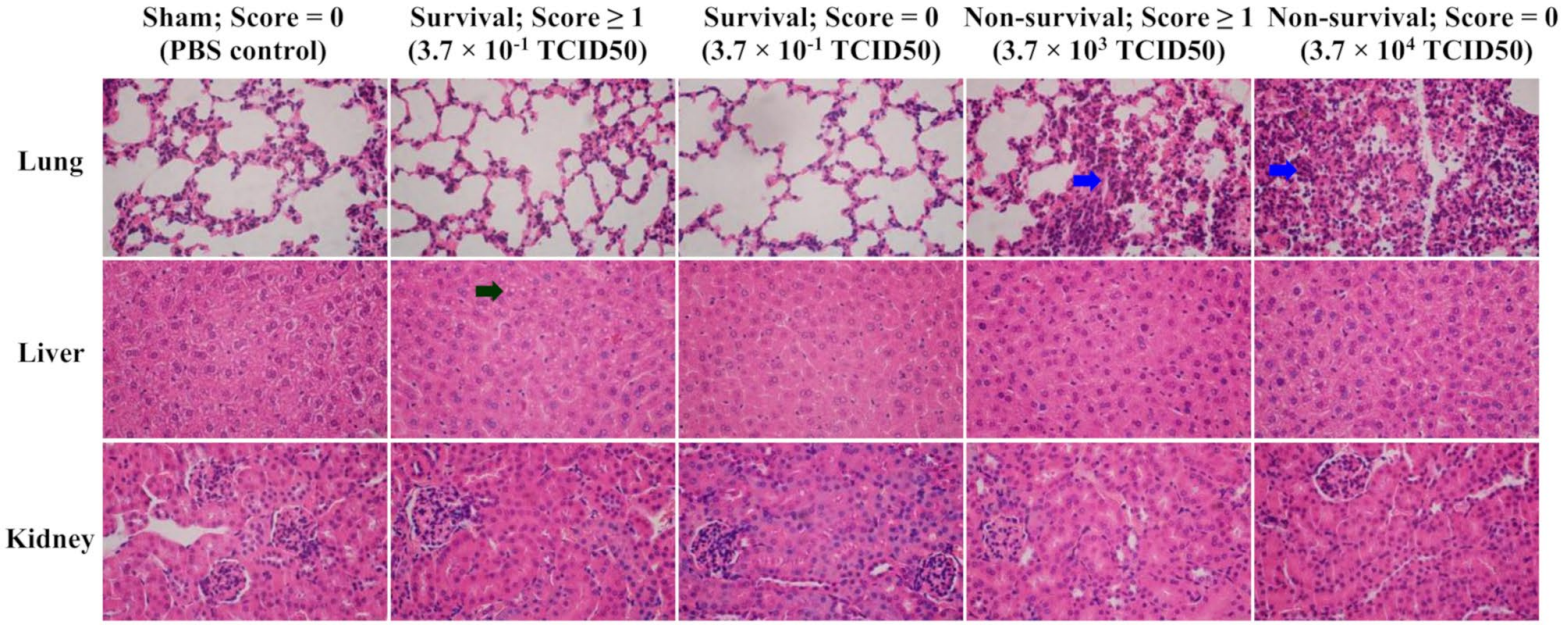
Histology of lung, liver and kidney from sham (PBS control) and influenza virus H1N1 strain A/PR/8/34 infected mice. Score = 0: no organ dysfunction; score ≥ 1: organ dysfunction detected by biochemistry tests. Histology was stained with hematoxylin and eosin (H&E). Blue arrow: massive inflammatory cellular infiltration; black arrow: hepatocytic vacuolation. Magnification: 600 ×. Size bar = 20 μm

Lung tissues displayed classic thin alveolar septa in sham controls and survived mice. Non-survived mice exhibited massive inflammatory cellular infiltration (Fig. 4). Acute lung injury appears to be the leading injury after H1N1 infection [23]. Hepatocytic vacuolation occurred in mice with detected liver dysfunction (Fig. 4), consistent with biochemistry results. No significant changes were seen in kidney, spleen, brain, or heart tissues (Supplementary file 6).

## Discussion

There is currently no gold standard murine sepsis model. We repurposed an influenza pneumonia model to assess its use for modelling viral respiratory sepsis. This strategy reduces time cost and is easy to implement. It will open an innovative way to develop experimental systems through collaborating with multiple research disciplines. We identified liver and renal dysfunction alongside lung inflammation in this model. This provides a prototype for building a murine model specific for viral respiratory sepsis, and more closely simulating human sepsis.

LPS models simulate endotoxemia, and surgery models, e.g., the caecal ligation and puncture (CLP) model, mimic abdominal sepsis [11]. This H1N1 model employs live influenza virus delivered intranasally, presenting a less-invasive and clinically relevant method for studying respiratory sepsis caused by viral infections. This model requires a higher biosafety level, which may limit its accessibility to some laboratories. Nevertheless, it serves as a valuable tool for advancing research into viral respiratory sepsis.

Mortality has been used to reflect sepsis, but although the mortality rate correlates with disease severity, it does not reflect organ dysfunction [24]. Further, sepsis does not necessarily lead to death. In fact, the majority of sepsis patients survive, with a mortality rate of 20–30% [25]. Hence, organ dysfunction could appear in survived mice but not must in non-survived mice. Bilirubin, creatinine, and platelets were used to quantify organ dysfunction, reflecting human SOFA scoring. In humans, the SOFA score is routinely used to monitor sepsis, reflecting the presence and severity of organ dysfunction [16]. Though MSS was developed to monitor murine sepsis, its subjective observational criteria do not address several critical components of human SOFA [14, 15]. There is a compelling need to develop a murine-SOFA comparable with human SOFA, which will allow for more confidence in translating animal study results into human studies.

Histological examination confirmed liver damage, consistent with liver dysfunction. No kidney damage was observed despite elevated serum creatinine indicating renal dysfunction biochemically. This aligns with reports of subtle histological changes in organs from septic mice despite significant biochemical changes [14]. In humans, it is impractical to perform biopsy to detect organ injury, resulting in a lack of histological evidence in sepsis patients.

One limitation of using this influenza model to produce viral respiratory sepsis is the selection of a dose that can lead to maximum levels of organ dysfunction in mice. There is no dose-response relationship observed between organ dysfunction and virus doses. Organ dysfunction appeared at lower doses but not consistently at the highest dose, and responses varied among mice inoculated with the same viral dose. Genetic susceptibility of individual mice to sepsis may contribute to the variability. This phenomenon mirrors clinical reality, where patients infected with influenza may experience varying disease severity; some develop sepsis, while others do not. These findings emphasise the need for future studies to determine an optimal dose of the H1N1 strain A/PR/8/34 to produce sepsis. Alternatively, a series of different doses will be required to produce this viral sepsis model, leading to difficulty in controlling experimental conditions, which will limit the use of this model in sepsis research.

The respiratory sepsis model will ultimately be used for biomarker discovery and drug research. The preclinical findings will be translated into human trials. Therefore, it is essential to determine the relevance of the experimental tools to real patients. Future studies will investigate the extent to which the mouse model reflects the real progression of viral sepsis in patients, and compare whether there is a similar progression trend of each parameter in SOFA scoring systems between mouse models and patients.

## Conclusions

This study demonstrates H1N1 influenza virus can cause organ dysfunction, providing a basis for building a murine model specific for viral respiratory sepsis and more closely simulating human sepsis. Larger-scale, dynamic studies are needed to refine and validate the performance of this model. A murine scoring tool comparable to SOFA is essential.

## Electronic supplementary material

Below is the link to the electronic supplementary material.

Supplementary Material 1

## Data Availability

All datasets, on which the conclusions of the manuscript rely on, are presented in the paper.

## Abbreviations

CI: Confidence interval
HEP: Humane endpoint
LPS: Lipopolysaccharide
MSS: Murine Sepsis Score
SD: Standard deviation
SOFA: Sequential Organ Failure Assessment
TCID50: Median tissue culture infectious dose
