# Supplementary material for: Use of H1N1 strain A/PR/8/34 influenza to build a mouse model of viral respiratory sepsis
Source: Lab Anim Res. 2025 Jun 4;41:16. doi: 10.1186/s42826-025-00248-4 (PMC12135557; doi:10.1186/s42826-025-00248-4)
Supplement: Supplementary file 1 — Supplementary Material 1 [file 42826_2025_248_MOESM1_ESM.docx]

**
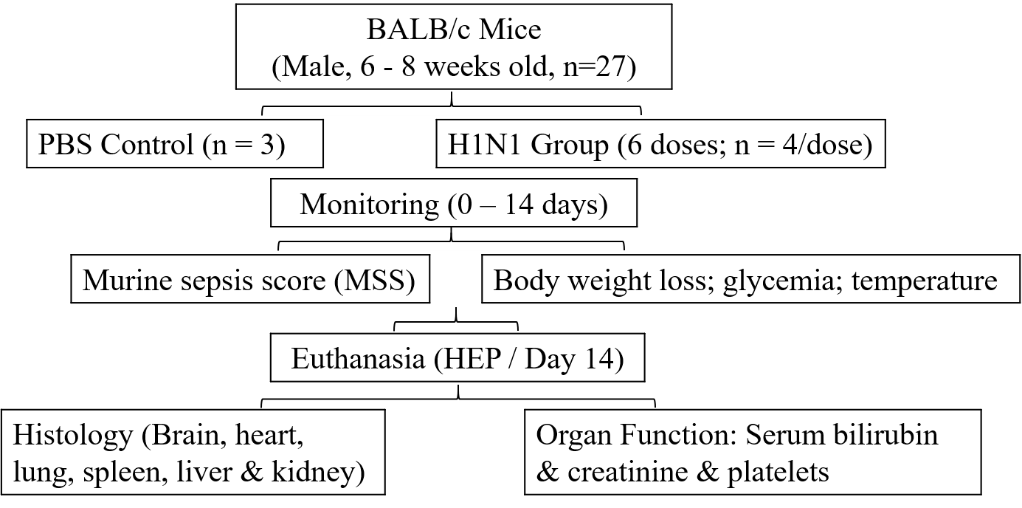
**

**Supplementary file 1** The workflow of assessing a mouse model infected with influenza H1N1 strain A/PR/8/34 to model viral respiratory sepsis.

**Supplementary file 2** Murine sepsis score (MSS).

| **Variable** | **0** | **1** | **2** | **3** | **4** |
| --- | --- | --- | --- | --- | --- |
| **Appearance** | Coat is smooth | Patches of hair piloerected | Majority of back is piloerected | Piloerection may or may not be present; mouse appears “puffy” | Piloerection may or may not be present; mouse appears emaciated |
| **Level of Consciousness** | Mouse is active | Mouse is active but avoids standing upright | Mouse activity is noticeably slowed. The mouse is still ambulant | Activity is impaired. Mouse only moves when provoked; movements have a tremor | Activity severely impaired. Remains stationary when provoked, with possible tremor |
| **Activity** | Normal amount of activity. Mouse is any of eating, drinking, climbing, running, and fighting | Slightly suppressed activity. Mouse is moving around bottom of cage | Suppressed activity. Mouse is stationary with occasional investigative movements | No activity | No activity. Mouse experiencing tremors, particularly in the hind legs |
| **Response to Stimulus** | Mouse responds immediately to auditory stimulus or touch | Slow or no response to auditory stimulus; strong response to touch (moves to escape) | No response to auditory stimulus; moderate response to touch (moves a few steps) | No response to auditory stimulus; mild response to touch (no locomotion) | No response to auditory stimulus. Little or no response to touch. Cannot right itself if pushed over |
| **Eyes** | Open | Eyes not fully open, possibly with secretions | Eyes at least half closed, possibly with secretions | Eyes half closed or more, possibly with secretions | Eyes closed or milky |
| **Respiration Rate** | Normal, rapid mouse respiration | Slightly decreased respiration (rate not quantifiable by the eye) | Moderately reduced respiration (rate at the upper range of quantifying by the eye) | Severely reduced respiration (rate easily countable by the eye, 0.5 s between breaths) | Extremely reduced respiration (>1 s between breaths) |
| **Respiration Quality** | Normal | Brief periods of laboured breathing | Laboured, no gasping | Laboured with intermittent gasps | Gasping |

**Supplementary file** **3** Human sequential organ failure assessment (SOFA) score.

| **System Parameters** | **0** | **1** | **2** | **3** | **4** |
| --- | --- | --- | --- | --- | --- |
| **Respiration** PaO2/FiO2, mmHg | Normal | < 400 | < 300 | < 200 (with respiratory support) | < 100 (with respiratory support) |
| **Coagulation** Platelets, x10^3^/µL | Normal | < 150 | < 100 | < 50 | < 20 |
| **Liver** Bilirubin, μmol/L | Normal | 20 – 32 | 33 – 101 | 102 – 204 | 204 |
| **Cardiovascular** Hypotension | Normal | MAP < 70 mmHg | Dopamine < 5 or dobutamine (any dose) | Dopamine > 5 or epinephrine < 0.1 or norepinephrine < 0.1 | Dopamine > 15 or epinephrine > 0.1 or norepinephrine > 0.1 |
| **Central Nervous System** Glasgow Coma Score (GSC) | Normal | 13 – 14 | 10 – 12 | 6 – 9 | < 6 |
| **Renal** Creatinine, μmol/L or Urine output | Normal | 110 – 170 | 171 – 299 | 300 – 440 or < 500 mL/day | > 440 or < 200 mL/day |

**Supplementary file 4** Proposed criteria of bilirubin, creatinine and platelets in assessing murine sepsis.

| **System Parameters** | **0** | **1** | **2** | **3** | **4** |
| --- | --- | --- | --- | --- | --- |
| **Liver** Bilirubin, μmol/L | Normal (0 – 28) | 29 – 46 | 47 – 144 | 145 – 290 | > 290 |
| **Renal** Creatinine, μmol/L | Normal (0 – 120) | 121 – 187 | 188 – 329 | 330 – 440 | > 440 |
| **Coagulation** Platelets, x10^3^/µL | Normal (≥ 1000) | < 1000 | < 667 | < 333 | < 133 |

Note: The ranges for each criterion were assigned by proportion corresponding to human SOFA.

**Supplementary file 5** Summary of individual mouse data across experimental groups and control group in H1N1 viral sepsis study.

| **Group** | **Mouse no.** | **MSS peak** | **Serum bilirubin, μmol/L** | **Serum creatinine, μmol/L** | **Platelet counts, x10^3^/µL** |
| --- | --- | --- | --- | --- | --- |
| **Sham (PBS control)** | **A1** | 0 | 6.96 | 35 | 1364; 1678 |
|  | **A2** | 0 | 20.66 | 90 | 1277; 1538 |
|  | **A3** | 0 | 4.83 | 23 | 1461; 1268; 1975 |
| **3.7 × 10^-1^ TCID50** | **B1** | 0.5 | 6.23 | 31 | 1314 |
|  | **B2** | 0 | 10.22 | 47 | 1476 |
|  | **B3** | 0 | 9.68 | 52 | 1278 |
|  | **B4** | 0 | 93.1 | 410 | 1247 |
| **3.7 × 10^0^ TCID50** | **C1** | 1 | 12.57 | 52 | ─ |
|  | **C2** | 1 | 39.37 | 171 | 1299 |
|  | **C3** | 1 | ─ | ─ | 1237 |
|  | **C4** | 0 | 37.07 | 166 | 1378 |
| **3.7 × 10^1^ TCID50** | **D1** | 0 | 8.68 | 41 | 2166 |
|  | **D2** | 0 | 36.49 | 161 | ─ |
|  | **D3** | 0 | 48.84 | 212 | ─ |
|  | **D4** | 1 | 9.11 | 36 | 1400 |
| **3.7 × 10^2^ TCID50** | **E1** | 2 | ─ | ─ | 1742 |
|  | **E2** | 0 | 8.79 | 43 | ─ |
|  | **E3** | 2 | 22.37 | 100 | ─ |
|  | **E4** | 2 | ─ | ─ | 1983 |
| **3.7 × 10^3^ TCID50** | **F1** | 6 | 18.22 | 78 | 1563; 1870 |
|  | **F2** | ~~4~~ | 18.29 | 88 | ─ |
|  | **F3** | 5 | 41.4 | 181 | 1819; 1469; 1040 |
|  | **F4** | 5 | 43.34 | 197 | 1715 |
| **3.7 × 10^4^ TCID50** | **G1** | 5 | ─ | ─ | 1043 |
|  | **G2** | 3 | 7.81 | 40 | 1516 |
|  | **G3** | 4 | 9.81 | 48 | 1815 |
|  | **G4** | 6 | 4.78 | 28 | 1698 |

Note: '-' indicates that the measurement was unavailable due to errors or insufficient blood volume for analysis.


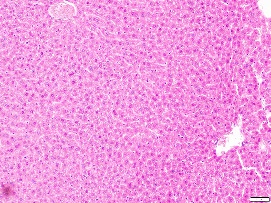


**Survival; Score ≥ 1**

**(3.7 × 10^-1^ TCID50)**


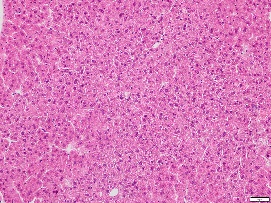

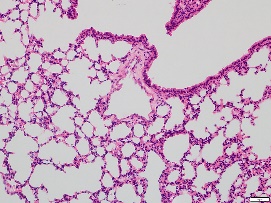


**Sham; Score = 0**

**(PBS control)**


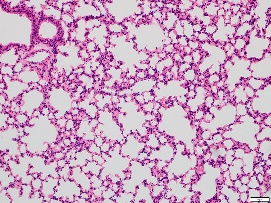


**Lung**

**Liver**

**Kidney**


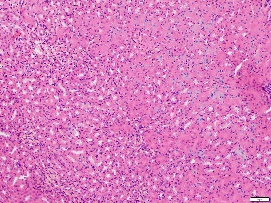

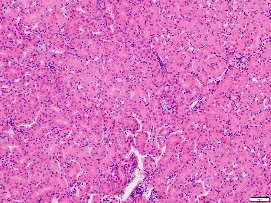


**Non-survival; Score ≥ 1**

**(3.7 × 10^3^ TCID50)**

**Survival; Score = 0**

**(3.7 × 10^-1^ TCID50)**


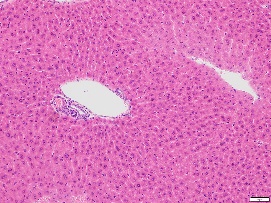

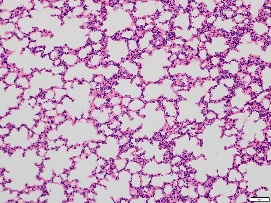

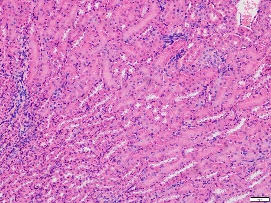

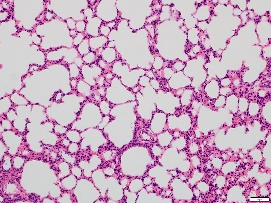

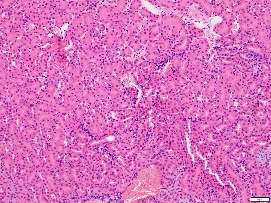

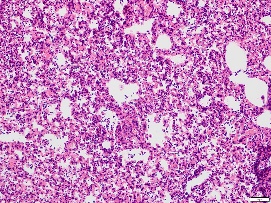

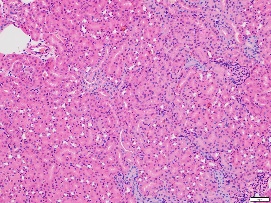


**Non-survival; Score = 0**

**(3.7 × 10^4^ TCID50)**


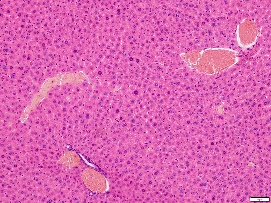

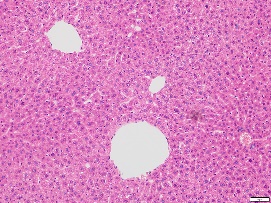

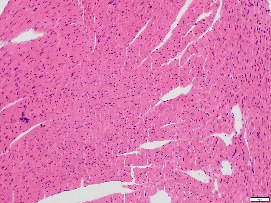

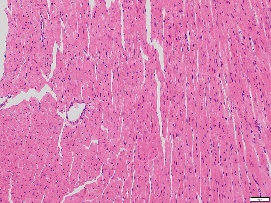

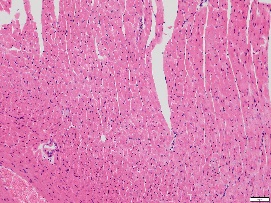

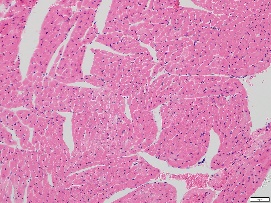

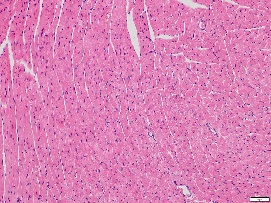

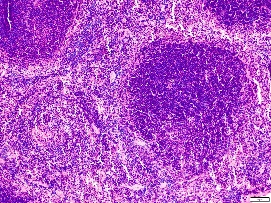

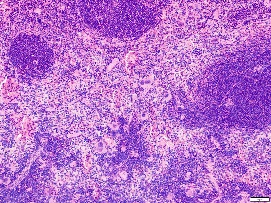

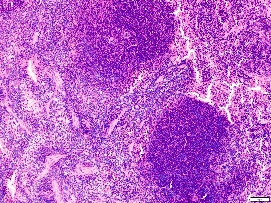

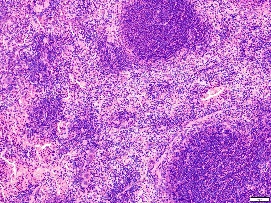

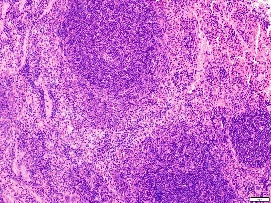

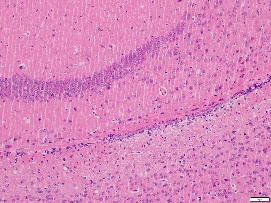

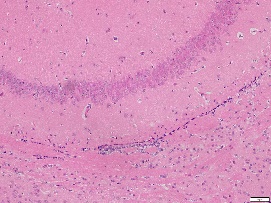

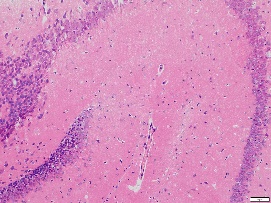

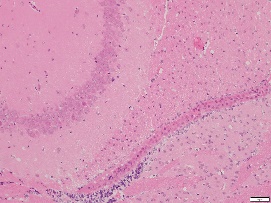

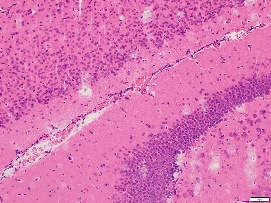


**Heart**

**Spleen**

**Brain**

**Supplementary file 6** Histology of lung, liver, kidney, heart, spleen and brain from sham (PBS control) and influenza virus H1N1 strain A/PR/8/34 infected mice. Score = 0: no organ dysfunction; score ≥ 1: organ dysfunction detected by biochemistry test. Histology was stained with hematoxylin and eosin (H&E). Magnification: 200 ×. Size bar = 50 µm.
